# Effect of protective glasses on radiation dose to eye lenses during whole breast irradiation

**DOI:** 10.1002/acm2.13073

**Published:** 2020-10-30

**Authors:** Tokiko Nakamura, Shoichi Suzuki, Kyoichi Kato, Napapong Pongnapang, Naoki Hayashi, Chie Kurokawa, Ikuo Kobayashi, Toru Negishi, Tamaki Matsunami

**Affiliations:** ^1^ Department of Radiology Juntendo University Shizuoka Hospital Shizuoka Japan; ^2^ Department of Radiological Sciences Tokyo Metropolitan University Graduate School of Human Health Science Tokyo Japan; ^3^ Department of Radiological Technology Fujita Health University School of Health Sciences Toyoake Aichi Japan; ^4^ Department of Radiological Technology Showa University Graduate School of Health Sciences Tokyo Japan; ^5^ Department of Radiological Technology Faculty Medical Technology Mahidol University Siriraj Hospital Bangkok Thailand; ^6^ Department of Radiological Technology Juntendo University School of Health Sciences Tokyo Japan; ^7^ Research Institute of Nuclear Engineering University of Fukui Tsuruga City Japan; ^8^ Nagase‐Landauer, Limited Tsukuba Japan

## Abstract

**Objectives:**

The efficacy of radiotherapy for breast cancer has greatly improved owing to better irradiation methods. Radiotherapy aims to deliver therapeutic doses to predetermined target volumes while sparing surrounding healthy tissues. However, there are few reports on radiation exposure to eye lenses, and the recommended exposure limits to ocular lens have been substantially reduced in recent years. This study aimed to investigate the amount of radiation exposure to eye lenses using optically stimulated luminescence dosimeters (OSLDs) and determine whether wearing special protective devices to protect the eyes, as an organ at risk, during whole breast irradiation, is necessary.

**Methods:**

This experiment used OSLDs on water‐equivalent phantom to measure the change in scattered radiation dose due to the difference of irradiation field while using 4‐ and 6‐MV photons of TrueBeam linear accelerator. Using a total treatment dose of 50 Gy, a target was positioned to approximate the breast, and a plan was formulated to deliver 2 Gy per treatment by tangential irradiation. The mean (SD) irradiation dose at the lens position outside the irradiation field was reported.

**Results:**

The scattered radiation dose outside the irradiation field was more affected by the irradiation field size than by the radiation energy. The out‐of‐field irradiation dose with a larger field of view was higher than that with a smaller field of view. The use of 0.07‐ and 0.83‐mm‐thick lead shield protective glasses reduced the radiation dose by 56.1% (*P* < .001) and 55.6% (*P* < .001), respectively.

**Conclusions:**

In this experimental model, the amount of radiation the eye was exposed to during whole breast irradiation was determined by the distance of the eye from the radiation field edge and by wearing protective glasses. In clinical practice, the protection offered by eyeglasses may reduce the risk of long‐term side effects and allow the use of higher intensive radiotherapy.

## 1 INTRODUCTION

With development of high‐precision radiotherapy in recent years, the number of applications of curative radiotherapy is increasing. Such developments in the irradiation technique, and its therapeutic effect, have increased the survival rate and improved prognosis. Substantial technical improvements have also provided multiple variants of radiation therapy capable of accurately delivering high therapeutic doses within specified target volumes while minimizing the exposure of radiosensitive organs to damaging and toxic effects of the treatment. In practice, the clinical teams optimize each treatment plan by considering the dose constraints (dose limits) for the defined organs at risk, for example, by using the Quantitative Analyses of Normal Tissue Effects in the Clinic (QUANTEC). Eye lenses have low tolerance to radiation, with increased risk for cataracts or lens opacification and secondary carcinogenesis.^1^, ^2^, ^3^, ^4^, ^5^ Cataract risk assessment is, therefore, important.^6^

In radiotherapy, there is a guideline for assessing and managing radiation doses to the area surrounding the treated region.^7^ The out‐of‐field radiation doses should be evaluated to be able to protect surrounding organs at risk from radiation and to guide treatment planning. However, there are difficulties in quantitatively assessing the effect of scattered radiation doses to organs at risk outside the irradiation field.

As advocated by the International Commission on Radiological Protection (ICRP),^8^ the lens of the eye is extremely sensitive to radiation. Although it is difficult to estimate the dose of scattered radiation to the lens of the eye, studies suggest that cataracts may occur at a much lower dose than previously considered.^9^ While discussions about exposure of the contralateral breast to radiation are ongoing, there are few reports on radiation exposure to the eye lenses, which are normal tissues near the breast, even though the tolerance dose of the lens (the dose that causes side effects in 5% of cataracts, requiring surgery in 5 years: TD_5/5_) is defined at 10 Gy in general radiotherapy. For occupational exposure, the ICRP recommends a dose limit for the lenses of 20 mSv/year, averaged over a 5‐year period, with not more than 50 mSv/year in any single year. For the general population, the limit is 15 mSv/year. The amount of radiation the lenses are exposed to need to be monitored due to the close proximity of the lenses to the irradiated area during radiotherapy of the breast.

Optically stimulated luminescence dosimeters (OSLDs) are important for measuring scattered radiation dose during interventional procedures, radiotherapy, and diagnostic radiology due to their small size, tissue equivalence, sensitivity, and reusability.^10^, ^11^, ^12^, ^13^ When performing measurements using OSLDs, it is necessary to confirm the characteristics of the dosimeter itself in order to accurately evaluate the air dose. The response characteristics of OSLDs to scattered radiation have been previously reported by Hirosawa et al. and are similar to those of ion chamber dosimeters.^14^ McKeever et al^15^ reported that OSLDs could measure doses as low as 10 μGy.

We conducted an experiment of the phantom that approximated the breast and lenses of eyes during whole breast irradiation and used OSLDs to determine the amount of radiation the eyes are exposed to during breast irradiation. We also evaluated the effect of wearing special protective devices to protect the eyes, as an organ at risk, to determine whether wearing protective devices is beneficial.

## 2 METHODS

### 2.1 Measurement of scattered radiation dose using OSLDs

**Fig. 1:**
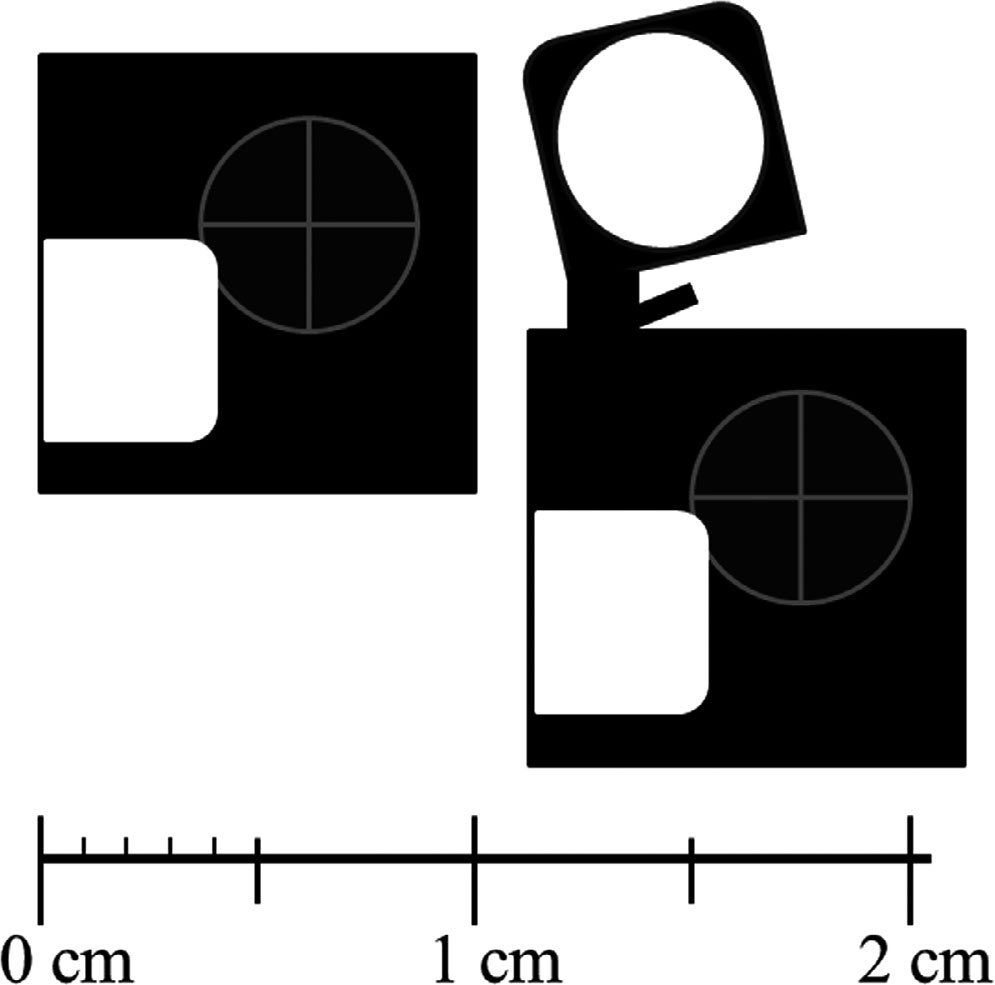
nanoDot optically stimulated luminescence dosimeter. The small‐type OSLD, called nanoDot, is carbon‐doped aluminum oxide with high luminous efficiency packed in a 10 × 10 × 2‐mm sized plastic disk.

The small‐type OSLD, known as nanoDot (Landauer Inc., Glenwood, IL, USA), is carbon‐doped aluminum oxide (α‐Al_2_O_3_: C) with high luminous efficiency packed in a 10 × 10 × 2‐mm‐sized plastic disk (Fig. 1). This device was used in this study along with its reader called the microStar reader (Landauer Inc., Glenwood, IL, USA).^16^

**Fig. 2:**
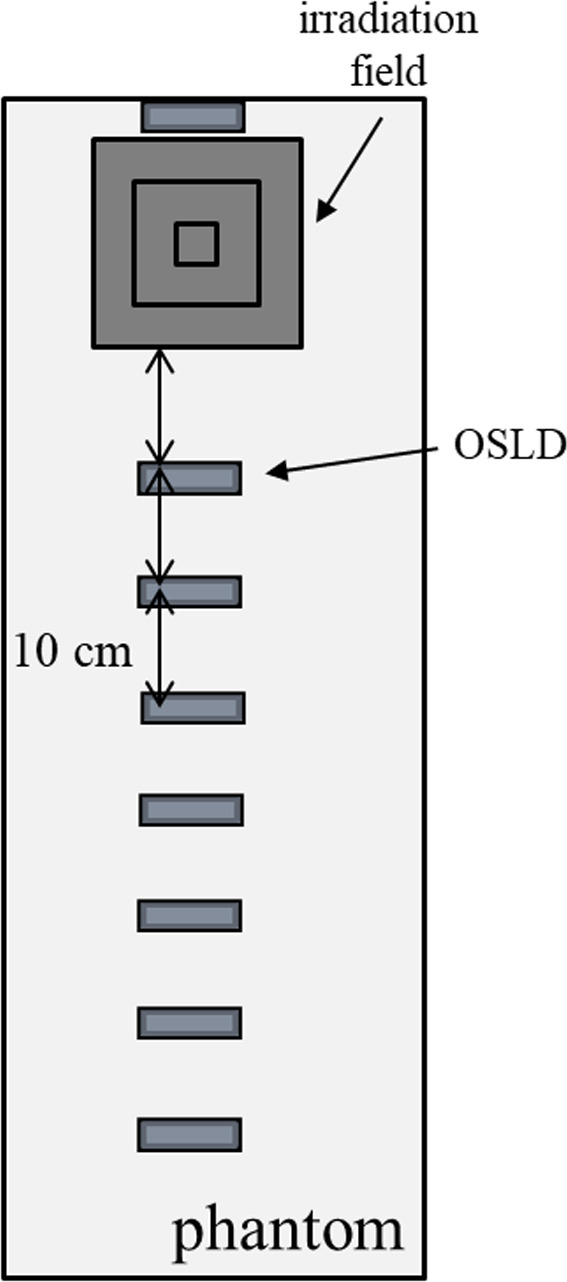
Dosimeter placement/layout. OSLDs placed on the water‐equivalent phantom at intervals of 10 cm from the irradiation fields edge to 70 cm.

A water‐equivalent phantom (ToughWater, Kyoto Kagaku Corporation) was placed in the longitudinal axis direction of the couch, and these disks were placed on a phantom and irradiated with 4‐ and 6‐MV photons of TrueBeam linear accelerator (Varian Medical Systems). The sizes of irradiation fields were 5 cm × 5 cm, 10 cm × 10 cm, and 20 cm × 20 cm. Fig. 2 shows OSLDs placed on the phantom at intervals of 10 cm from the irradiation fields edge to 70 cm. The number of repetitions performed for each measurement was 10. After the x‐ray irradiation, the reading was performed five times each, and the air kerma was calculated using a formula (1). The background value was the value of the element not irradiated. We also calibrated OSLDs with a 4‐MeV x ray at a depth of 10 cm in the primary beam.

(1) $$\text{Dose}\left(\text{mGy}\right)=\text{OSLDs}/\text{reader calibration constant}\left(\text{counts}/\text{mGy}\right)\times \text{OSLDs sensitivity}$$

### 2.2 Dosimetry at the position of the detectors meant to represent the human eye lens

**Fig. 3:**
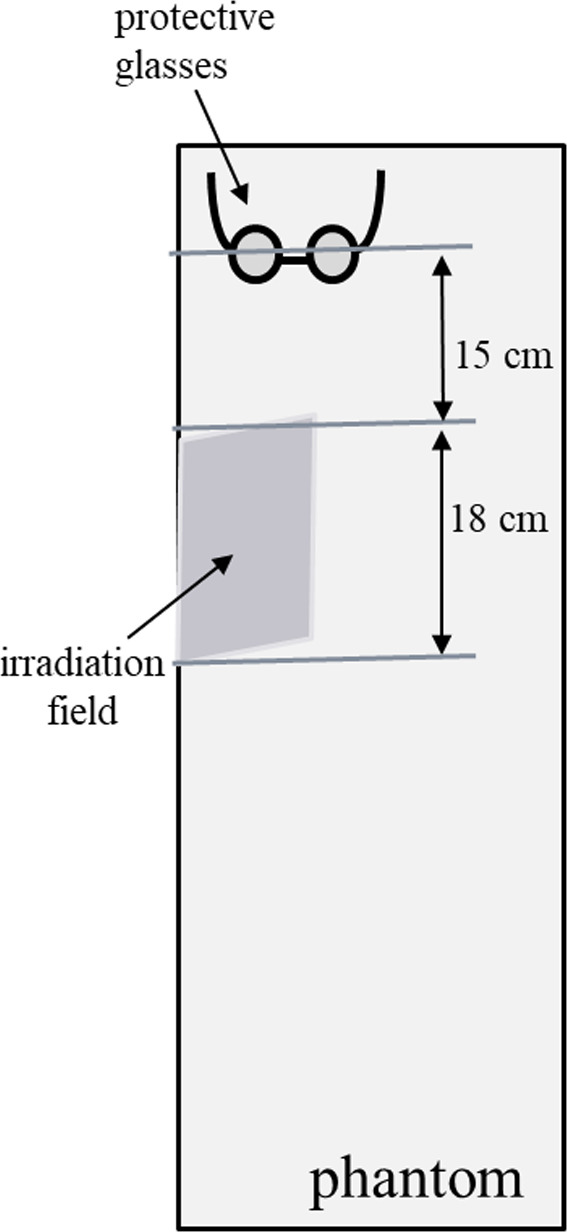
Dosimeter placement/layout. A water‐equivalent phantom was placed in the longitudinal axis direction of the couch, and the target area was defined as the position that assumed the breast area in the phantom.

A water‐equivalent phantom was placed in the longitudinal axis direction of the couch, and the target area was defined as the position that assumed the breast area in the phantom (Fig. 3). The initial prescribed plan was to deliver 2 Gy using 6‐MV x‐ray irradiation (TrueBeam linear accelerator) to the target area (18 cm in length) by the tangential irradiation technique. The value measured by irradiating 2 Gy was multiplied by 25 to obtain the 50‐Gy total planned dose. OSLDs were placed horizontally at the estimated position of the detectors meant to represent the human eye lens, which was 15 cm from the edge of the field. The doses were measured using protective glasses, normally used by radiation medical staff, containing 0.07‐ and 0.83‐mm equivalent leads, to compare the difference in the dose with and without protective glasses. The number of repetitions performed for each measurement was 10. The measured values were compared to the international guidelines from ICRP 138.^17^

### 2.3 Statistical analysis

The scattered dose was expressed as mean and standard deviation. All statistical analyses of the recorded data were performed using the Excel statistical software package (Ekuseru‐Toukei 2015; Social Survey Research Information Co., Ltd., Tokyo, Japan).

## 3 RESULTS

### 3.1 Measurement of scattered radiation dose by OSLDs

**Fig. 4:**
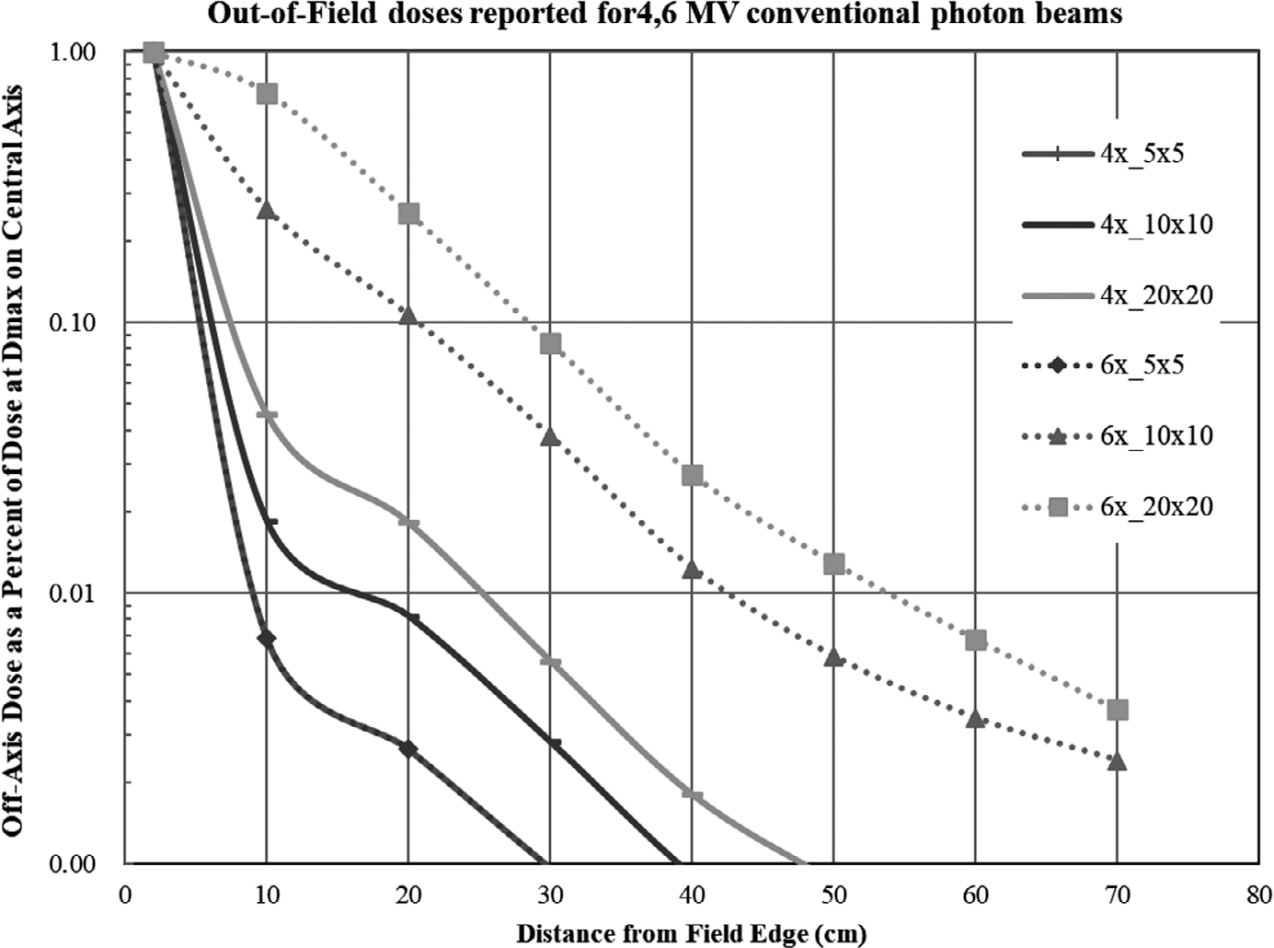
Dose outside the irradiation field.

The results are shown in Fig. 4. Regardless of photon energy, dose attenuation was confirmed as a function of distance from the radiation field edge (*P* < .001). The dose from 4‐MV irradiation to a planned dose of 2 Gy with a 5 cm × 5 cm field was 3 ± 0.1 mGy at the 20‐cm off the field edge. For the 10 cm × 10 cm and 20 cm × 20 cm fields, the doses were decreased to 11 ± 0.5 mGy and 25 ± 0.2 mGy, respectively. At the 20‐cm point, doses from 6‐MV irradiation with 5 cm × 5 cm, 10 cm × 10 cm, and 20 cm × 20 cm fields were 40 ± 1.5 mGy, 136 ± 1.7 mGy, and 336 ± 4.3 mGy, respectively. It was observed that the out‐of‐field dose with a larger field of view was higher than the dose irradiated with a smaller field of view (*P* < .001). At 6‐MV energy, the radiation field was attenuated to a dose of 1/100 at 30/40/50 cm or more from the periphery of the irradiation field at 5 cm × 5 cm, 10 cm × 10 cm, and 20 cm × 20 cm. The result of 4‐MV irradiation showed a similar trend. The dose outside the irradiation field was found to be more affected by the irradiation field size than the energy.

### 3.2 Dosimetry at the lens of the eye

As shown in Table 1, the cumulative dose at the detectors meant to represent the human eye lens position during 50‐Gy irradiation of the breast was 307.6 ± 7.1 mGy without protective glasses. The dose decreased to 172.8 ± 3.2 mGy (56.1% reduction, p < 0.001) when using 0.07‐mmPb protective glasses and 171.1 ± 6.2 mGy (55.6% reduction, *P* < .0001) when using 0.83‐mmPb protective glasses. There was no difference between doses with 0.07‐mmPb and 0.83‐mmPb protective glasses (*P* = .37).

**Table 1:** Cumulative dose at detectors meant to represent the human eye lens position during 50‐Gy irradiation of the breast

	Without glasses	With protective glasses	
		0.07 mmPb	0.83 mmPb
Cumulative dose	307.6 ± 7.1 mGy	172.8 ± 3.2 mGy	171.1 ± 6.2 mGy

## 4 DISCUSSION

This study demonstrates that regardless of photon energy, the radiation dose is attenuated as a function of distance from the radiation field edge and that the use of protective glasses reduces the cumulative dose of radiation the eye lenses are exposed to during breast irradiation.

When using OSLDs in the high‐energy region, such as those used in radiation therapy, the size of the detector can be a disadvantage, although transmission of the radiation to the detector can be ignored. However, as with other detectors, dose evaluation can be performed correctly as long as calibration is performed. The OSLDs used in this experiment have a maximum variation of ±5% during device manufacturing. This is also clear from the work of Al‐Senan et al.^18^ Therefore, it is considered that the variation of the OSLD did not affect the dose evaluation.

The OSLDs measured total scatter radiation, which included linac head scatter, scattering from the phantom, etc. However, we believe that linac head scatter radiation was negligible due to the distance between the linac edge and eye lens. As demonstrated in Fig. 4, there was an exponential dose reduction from field edge to distal measurement points. Similarly, in a study using Monte Carlo simulations, Wijesooriya, et al. reported that out‐of‐field doses reduced exponentially with distance.^19^ Overall, these results demonstrate the feasibility of using OSLDs for low‐scattered radiation measurements. In addition, we believe that neutron creation was negligible because we used a <6‐MV photon beam for whole breast irradiation.

The tolerable dose of the detectors meant to represent the human eye lens in radiation therapy, TD_5/5_ of eye lens is defined at 10 Gy, so the dose constraint was satisfied. The ICRP and the Seoul statement^8^ defines dose limits that are lower than cataract threshold doses. Radiation dose, in the range of one to several tens of Gy, causes tissue damage, and at higher doses, hematopoietic and digestive dysfunction may cause acute symptoms or death. High doses can lead to chronic dysfunction and carcinogenesis after decades. The effects on these tissues do not occur if the radiation dose is kept below a certain dose. This is called a dynamic effect. Since tissue dysfunction does not occur at doses of a few hundred mGy, only the risk of long‐term latent carcinogenesis is considered in this dose range. However, radiation‐induced cancer and genetic effects, which are stochastic effects, are late‐onset. In addition, optimization of protection for patients is unique. In the first place, radiation therapy is entirely different from anything else in that the dose to a human being is intentional and its potentially cell‐killing properties are the very purpose of the treatment. In such cases, optimization becomes an exercise in minimizing doses (and/or their deleterious effects) to surrounding tissues without compromising the predetermined and intentionally lethal dose and effect to the target volume.

**Fig. 5:**
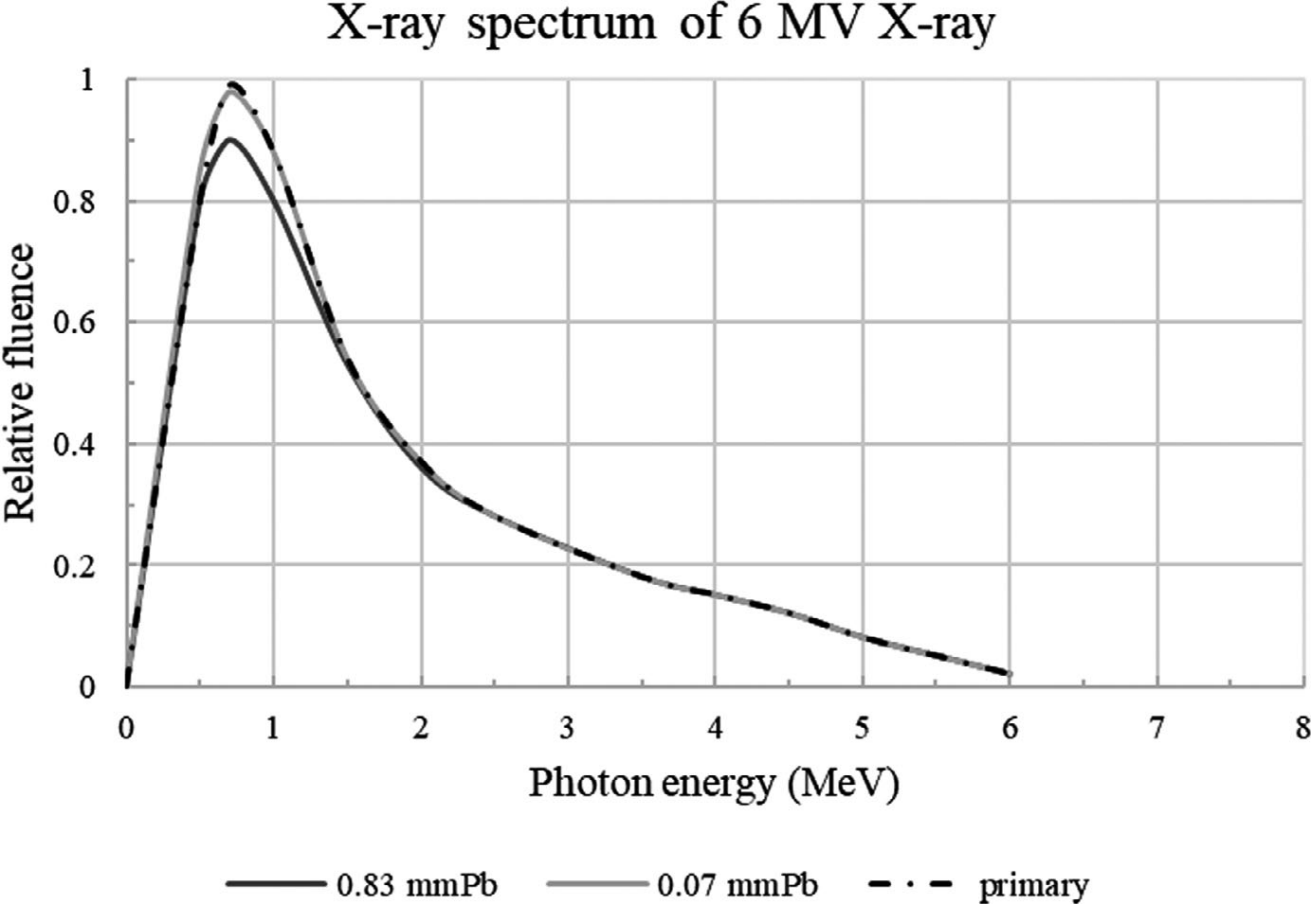
Six‐megavolt x rays irradiated during Monte Carlo simulation calculation; transmittance when using 0.83‐mmPb and 0.07‐mmPb protective glasses. 6‐MV x‐ray Calculation using Schiff's formula, Tungsten target, Average thickness of flattening filter = 6.5 mm.

Although the relationship between cumulative dose and irradiation period has not been confirmed, wearing protective glasses at the time of treatment may prevent development of late‐onset cataracts. In particular, since a large number of the patients undergoing breast radiotherapy are younger, the principle of radiation protection should be adhered to; benefit should exceed risk of harm, and radiation exposure dose should be kept as low as possible (as low as reasonably achievable, ALARA).^20^ In addition, determining the amount of radiation received by the detectors meant to represent the human eye lens during breast irradiation helps improve the radiation protection protocol and creates an optimal treatment plan for the patient. This was demonstrated by the difference in scattered doses when using protective glasses. However, when performing Monte Carlo simulation calculations, 6‐MV x rays were irradiated, and transmittance was at 93.3% when using 0.83‐mmPb protective glasses and 99.4% when using 0.07‐mmPb protective glasses. Only 7% and 1% were blocked, respectively (Fig. 5). In the high‐energy region, if the peak energy does not change for scattered direct radiation, the attenuation ratio should be theoretically the same. However, the actual measurement is different because the scattered radiation is not separated into scattering from the phantom and scattering from the head, that is, scattering behavior is unclear.^21^, ^22^ Therefore, the present measurement result shows that the scattered radiation dose at the position of the eye lens decreases only by wearing the glasses and cannot be shown by the apparatus and system for planning treatment. In future, it will be possible to accurately measure the exposure dose outside the irradiation field due to dose lines, and it will be necessary for patient dose management.

This experiment should be interpreted considering the following limitations. The study was unable to evaluate the long‐term effects of irradiation on the eye lenses when protective equipment was used. The physiological ability of the lens to repair was also not considered. Animal studies and long‐term human clinical trials are needed to validate these findings, taking into consideration the limitations highlighted in this experiment.

## 5 CONCLUSION

In this experimental model, the amount of radiation the eye was exposed to during whole breast irradiation was determined by the distance of the eye from the radiation field edge and by wearing protective glasses. In clinical practice, the protection offered by eyeglasses may reduce the risk of long‐term side effects and allow the use of higher intensive radiotherapy if necessary.

## CONFLICT OF INTEREST

No conflict of interest.
